# Evaluation of a Fecal Shedding Test To Detect Badger Social Groups Infected with Mycobacterium bovis

**DOI:** 10.1128/JCM.01226-20

**Published:** 2020-12-17

**Authors:** Andrew R. J. Murphy, Emma R. Travis, Victoria Hibberd, David Porter, Elizabeth M. H. Wellington

**Affiliations:** a School of Life Sciences, University of Warwick, Coventry, United Kingdom; University of Tennessee at Knoxville

**Keywords:** badger, bovine tuberculosis, disease reservoir, *Mycobacterium bovis*, cattle, environmental microbiology, molecular epidemiology

## Abstract

Bovine tuberculosis (bTB) is an economically important disease affecting the cattle industry in England and Wales. bTB, caused by Mycobacterium bovis, also causes disease in the Eurasian badger (Meles meles), a secondary maintenance host. Disease transmission between these two species is bidirectional. Infected badgers shed M. bovis in their feces. The Animal and Plant Health Agency (APHA) of the United Kingdom organized a comparative trial to determine the performance of tests in detecting M. bovis in badger feces for the Department for Environment, Food, and Rural Affairs (DEFRA). Here, we assessed the performance of the existing Warwick Fast24-qPCR test and its modified version based on a high-throughput DNA extraction method (Fast96-qPCR). We found Fast24-qPCR to have a sensitivity of 96.7% (95% confidence interval [CI], 94.5 to 99%; *n* = 244) and a specificity of 99% (95% CI, 97.8 to 100%; *n* = 292). Fast96-qPCR requires further optimization. Determining the disease status of badger social groups requires multiple tests per group. Therefore, to increase specificity further, we independently repeated the Fast24-qPCR test on positive samples, increasing stringency by requiring a second positive result. Fast24-qPCR with repeat testing had a sensitivity of 87.3% (95% CI, 83.1 to 91.5%; *n* = 244), and a specificity of 100% (95% CI, 100 to 100; *n* = 201) on an individual-sample level. At the social-group level, this repeat testing gives Fast24-qPCR high herd specificity, while testing multiple samples per group provides high herd sensitivity. With Fast24-qPCR, we provide a social-group-level test with sufficient specificity and sensitivity to monitor shedding in badgers via latrine sampling, delivering a potentially valuable tool to measure the impacts of bTB control measures.

## Abstract (precis)

Bovine tuberculosis (bTB) is an economically important disease affecting the cattle industry in England and Wales. bTB, caused by Mycobacterium bovis, also causes disease in the Eurasian badger (Meles meles), a secondary maintenance host. Disease transmission between these two species is bidirectional. Infected badgers shed M. bovis in their feces. The Animal and Plant Health Agency (APHA) of the United Kingdom organized a comparative trial to determine the performance of tests in detecting M. bovis in badger feces for the Department for Environment, Food, and Rural Affairs (DEFRA).

## INTRODUCTION

Bovine tuberculosis (bTB) caused by Mycobacterium bovis is an economically important disease that is estimated to cost £100 million to the taxpayer per year (1) in the United Kingdom. Prevalence of the disease in cattle herds has increased from 0.49% in 1979 (1) to 5.3% in England and 5.6% in Wales in 2019 (2), though it is not evenly distributed and is concentrated in the high-risk area of southwestern England and the surrounding edge area, in addition to southwestern and eastern Wales. In Britain, the Eurasian badger (Meles meles) is considered a secondary maintenance host of bTB (3), with an estimated bTB prevalence of 24.2% (4) within the high-risk area of England. Although cattle-to-cattle aerosol transmission is considered the predominant route of infection (5), badgers are known to transmit disease to cattle (6, 7) and are estimated to contribute up to 52% of individual cases within areas of endemicity (8), inclusive of subsequent cattle-cattle transmission. Cattle are also implicated in the transmission of bTB to badgers, as delays in removing infected cattle have been shown to increase bTB prevalence in badgers (7).

The route of transmission between badgers and cattle has not been demonstrated. However, several lines of evidence suggest an important role for transmission occurring through contamination of the environment. Direct contact between badgers and cattle is a rare event (9, 10), and cattle-cattle and badger-badger transmission rates are also low (11–13), despite high levels of intraspecies contact in these social animals. This suggests that the levels of direct contact are not high enough to explain the levels of interspecific transmission. Although badgers avoid direct contact with cattle (14), they actively favor cattle pasture for foraging, suggesting common occurrence of shared environment. M. bovis has been shown to persist in the environment in a number of studies (15–17), and models of bTB transmission in cattle have also suggested a substantial role for the environment (18) as a reservoir and route of transmission.

Various studies have also implicated the environment as a vector for bidirectional M. bovis transmission between cattle and badgers. Badgers sampled from a natural population shed M. bovis cells in sputum, urine, and feces (19, 20), indicating that contamination of pasture with feces and urine creates a potential source of infection (21). Furthermore, cattle do not avoid areas contaminated with badger urine and will graze at badger latrines given sufficient competition for fresh pasture (22). Similarly, the feces of infected cattle contained viable M. bovis (23, 24), though cattle were not shown to be infected from pastures contaminated with these feces, and, as these experiments were performed in the 1930s, it cannot be assumed that disease progression occurs in the same fashion under modern bTB testing regimens. However, at the point of detection by the single intradermal comparative tuberculin test (SICCT), the test used in the United Kingdom to detect bTB in cattle (2), the disease has progressed to the extent that 55.5% of positive animals have visible lesions at slaughter (25), compared to a background rate of 0.63/1,000 (0.063%) in negative animals (26). The spreading of slurry is also considered a risk factor for bTB breakdowns (27). Finally, badgers are known to forage under cattle dung (28), and earthworms have been demonstrated to spread M. bovis BCG from spiked cattle feces to surrounding soil (29).

A number of assays have been developed to diagnose bTB infection in badgers. These include immunoassays such as gamma interferon (IFN-γ) assays (4) and the BrockTB Stat-Pak assay (4), as well as culture from clinical samples (19, 30, 31). Sensitivity (Se) estimates of IFN-γ assays range from 52 to 85% in adult badgers (4, 31–33)—estimates for cubs are lower—compared with a range of 49 to 78% (depending on the severity of disease) for Stat-Pak (34). Specificity (Sp) estimates for IFN-γ range from 88 to 94% (4, 31, 32), compared with 93 to 97% for Stat-Pak (31, 34). Culture is very insensitive (8%), though it is considered to have near perfect specificity (99.8%) (31). These tests are not considered sensitive or specific enough to use alone and also necessitate the live trapping of animals, which requires intensive effort to achieve high coverage and is expensive (35, 36). It has been suggested that diagnosis should be performed at the social-group level, using IFN-γ and Stat-Pak assays in parallel (33). However, in order to maintain sufficient specificity at the group level, this approach requires a threshold of 2 badgers with positive tests to accurately identify a social group as infected. Therefore, this approach requires substantial trapping coverage (50%) and is unlikely to identify social groups with only one positive animal. The prevalence of M. bovis shedding in badger feces correlates well with prevalence of infection, as determined by IFN-γ and Stat-Pak assays of contemporaneous trapped badgers at a social-group level (37), though social groups with similar prevalence of infection showed heterogeneity in prevalence of shedding (38).

A culture-independent quantitative PCR (qPCR) test for detecting M. bovis DNA in environmental samples, including badger feces, was developed at Warwick University (39, 40). Pathogen detection in feces was thus pioneered by the use of qPCR on DNA extracted from feces and soil. It was possible to culture M. bovis from a proportion of qPCR positive badger feces (20), indicating pathogen viability; however, not all qPCR-positive feces provided culture-positive data due to the low sensitivity of culture from fecal samples. Quantification of the level of M. bovis genome equivalents in badger feces is likely to be a good proxy for the status of shedding through sputum, and thus transmission of infection through the respiratory route and biting; because lesions in the gut of badgers are extremely rare (41), the presence of M. bovis DNA in feces likely occurs via the passage of infected lung discharge through the gastrointestinal tract. Indeed, the detection of M. bovis DNA in both the trachea and feces of infected badgers correlates with severity of disease (42). The test also has the advantage of being noninvasive, with the potential to provide greater coverage of the population than trapping-based methods.

In this study, we assessed the diagnostic sensitivity and specificity of Fast24-qPCR, our existing DNA extraction and qPCR method, and Fast96-qPCR, a novel high-throughput DNA extraction method. Fast96-qPCR uses the same qPCR but a high-throughput version of the DNA extraction based on the same chemistry as Fast24-qPCR. We demonstrate that Fast24-qPCR provides a noninvasive method to detect bTB-infected badger social groups through latrine sampling with a high degree of social-group-level specificity and sensitivity. This will provide a valuable tool to enable monitoring of badger social-group bTB status through M. bovis shedding in badger feces and, by extension, the effects of bTB control measures.

## MATERIALS AND METHODS

### Production of the panel.

In order to determine the diagnostic sensitivity and specificity of the tests involved in this comparative study, a panel consisting of spiked positive samples (*n* = 245), known negative samples (*n* = 205), and putative positive samples (*n* = 119) from 12 badger social groups containing badgers known to be positive by serological testing was prepared by the Animal and Plant Health Agency (APHA). APHA required minimum acceptable thresholds of social-group-level sensitivity and specificity (50% and 80%, respectively), on behalf of the Department for Environment, Farming and Rural Affairs (DEFRA). These thresholds are based on the assumption of a 10% within-herd prevalence (in badgers) and 10 samples analyzed per social group, and as such, they require a sample-level (diagnostic) sensitivity of 50% and specificity of 98%. The numbers of known positive and negative feces in the panels were thus calculated to be able to establish these thresholds, with a 5% margin of error within 95% confidence intervals in the case of sensitivity and a 2% margin of error within 95% confidence intervals in the case of specificity (43). This required minimums of 246 known positive samples and 188 known negative samples. All study participants were blinded to the status of all samples within this panel until the completion of testing. APHA is compliant with the Animals (Scientific Procedures) Act 1986, and in addition, all experiments involving animals are both reviewed and approved as well as subjected to retrospective analysis by an ethics committee composed of veterinarians, animal care staff, a biostatistician, scientists, and lay members of the community. The production of the panel is as follows.

Feces (*n* = 50) were collected from badgers of known negative status at APHA Weybridge (*n* = 25) and APHA York (*n* = 12) and also from wild latrines at APHA Woodchester (*n* = 10) and latrines in regions of the country where bovine TB is not endemic in cattle (*n* = 3). Fecal samples were collected from a variety of sources in order to account for factors such as fecal consistency and presence of inhibitors, as any test used on wild samples must be robust to these factors. APHA personnel conducted the sampling and prepared spiked samples as follows: positive fecal samples (*n* = 245) were prepared by spiking 150 g pooled from the above sources with 20 ml of buffer containing known quantities (10^5^ to 10^1^ CFU/g) of M. bovis 2122/97. To ensure that spiked samples were homogenous, the feces were mixed with a spatula for a period of 5 min. Aliquots of 1 g were then frozen at −20°C in 2-ml screw-cap microcentrifuge tubes. Five 1-g aliquots were taken from each spiked 150-g fecal sample; thus, there were 5 technical replicates of 49 biological replicates. Full details of the protocol used can be found in the DEFRA report (44). The dilution series chosen was based on previous research within our group at Warwick University (37, 38), and concentrations were determined based on CFU count.

Negative feces (*n* = 205) were aliquoted in a separate laboratory to prevent contamination; feces were mixed with a spatula for 5 min, and aliquoting was performed as described above. Five of these negatives were prepared by spiking feces as described above with dilution buffer only.

Putative positive samples were taken from latrines connected to 12 historically positive social groups at APHA Woodchester. These social groups had at least one culture-positive result and/or four positive IFN-γ or BrockTB StatPak test results on trapped animals within the 2-year period preceding the sampling. Though this does not guarantee that the social groups still contained infected animals at the time of sampling, it was considered a reasonable assumption. The aim was to sample 10 scats per social group; however, only 9 were available from one social group. Therefore, the panel contained 119 putative positive samples. Approximately 30 g of each scat was mixed with a spatula for 5 min. One 1-g aliquot was taken from each unique scat to make up the panel, as described above.

Samples were deidentified by APHA, and two replicates of the panel (one for each extraction method) were sent to Warwick University using the appropriate secure transport procedures (WHO shipping number UN 2814).

In addition to the deidentified samples in the panel, known negative feces (*n* = 88) were added at Warwick University. These feces were also collected from badgers of known negative status at APHA Weybridge. This was to provide our own internal indicator of test performance. The composition of the panel is presented in Table 1.

**TABLE 1:** Composition of the panel used in this study^*a*^

Spiked M. bovis concn (CFU/g)	No. of samples
570,666.7	5
114,000	25
57,066.67	25
11,400	25
5,706.67	20
1,140	25
570.67	25
285.33	30
142.67	25
71.33	15
35.67	15
17.83	10
0	5
Negative (as part of original panel)	200
Negative (added at Warwick)	88 (Fast24-qPCR), 24 (Fast96-qPCR)
Putative positive	119

a The four lowest spiked concentrations (17.83 to 142.67 CFU/g) were used to determine diagnostic sensitivity at low concentration (DSeLC).

### DNA extraction from badger feces.

DNA was extracted from the badger feces using two methods, the existing Fast24-qPCR extraction and the new Fast96-qPCR extraction. For Fast24-qPCR, total community DNA was extracted from 0.1 g (±0.005 g) of feces using the FastDNA spin kit for soil (SKU 116560200-CF; MP Biomedicals) per the manufacturer’s instructions. A modified ribolysis step involving two sequential homogenization steps of 40 s at 6,000 rpm separated by a 30-s pause was performed using a Precellys 24 homogenizer (P000669-PR240-A; Bertin Instruments) as previously reported (37). DNA was extracted from the Fast24-qPCR panel twice in parallel by two operators.

For the Fast96-qPCR extraction, total community DNA was extracted from 0.1 g of feces using the FastDNA 96 soil microbe DNA kit (SKU 119696200; MP Biomedicals) with some alterations from the manufacturer’s instructions, detailed as follows. Ribolysis took place in lysing matrix E tubes (SKU 116914050-CF; MP Biomedicals) containing 400 μl lysis buffer and 100 μl sterile molecular-grade distilled H_2_O (dH_2_O). Samples were ribolysed using a FastPrep-96 instrument (SKU 116010500; MP Biomedicals) at 1,600 rpm for 60 s and centrifuged at 16,110 × *g* for 10 min. The supernatant was transferred to a 96-well deep-well plate, and DNA extraction then continued as per the manufacturer’s instructions. All subsequent centrifugation steps were performed in an Eppendorf 5810R instrument using the A-2-DWP-AT rotor at 3,486 × *g*. DNA was extracted from the Fast96 panel once.

### qPCR testing.

The RD4 qPCR assay was used as described previously (37). Briefly, samples were screened using duplicate qPCR assays, and those with a positive result in either replicate were subjected to full quantification in triplicate. A serial dilution of genomic DNA from M. bovis BCG Danish 1331 was used as the standard. If one or more replicates showed amplification in the quantification assay, then samples were deemed positive; otherwise, samples were deemed negative. Assays were performed for inhibition of the qPCR using a previously described inhibition assay (40) according to the previously described protocol (37), in order to detect possible false negatives. Briefly, an inhibition control was previously designed with an exotic probe target (green fluorescent protein [GFP]) flanked by DNA complementary to the RD4 primers. A known concentration of this target was added to all samples; if inhibitory compounds were present in the sample, qPCR of the inhibition control target was impacted in comparison with the negative control (equivalent volume of dH_2_O). This was quantified by comparison of the threshold *C_T_* of each sample to a negative control, with the difference in *C_T_* values referred to as Δ*C_T_*. Samples were screened in singlet, and if *C_T_* differed by >2.5 from that of the negative control, the sample was rescreened in duplicate. If the average *C_T_* of the duplicates differed by >2.5 from the negative control, the sample was considered inhibited. Inhibited, nonpositive samples were excluded from analysis. All qPCRs were performed in an ABI 7500 Fast qPCR system (4351106; Thermo Fisher Scientific), using 10 μl of DNA as the template. qPCR protocols were identical for both extraction methods.

### Statistical analysis.

**FIG 1:**
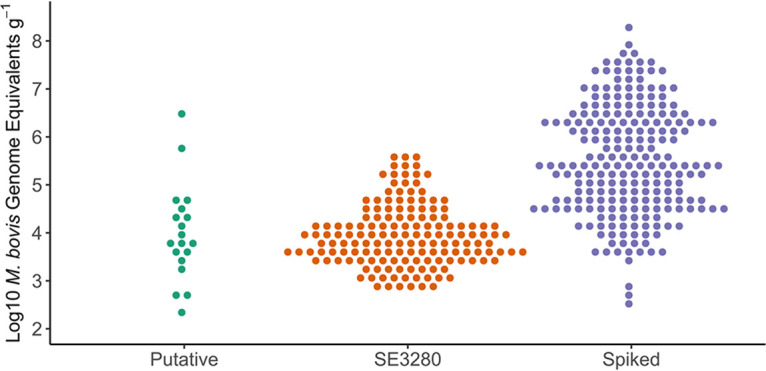
Log_10_ distribution of M. bovis genome equivalents obtained by qPCR of positive samples and comparison to a standard curve. Putative and spiked samples are from this study; SE3280 samples are from a previous DEFRA project (SE3280) reported by King et al. (37, 38). Putative and SE3280 samples are taken from natural infected populations. One-way analysis of variance (ANOVA) shows a significant difference between the means of the three populations (*P* < 0.001). Bonferroni-corrected two-tailed Welch’s *t* test shows significant differences between SE3280 and spiked samples (*P* < 0.001) and between putative and spiked samples (*P* < 0.001) but not between SE3280 and putative samples (*P* = 0.90).

The study was designed to assess the performance of multiple tests in detecting M. bovis in badger feces. Performance was measured in terms of diagnostic sensitivity and specificity at the individual sample level (DSe and DSp). Based on discrepancies between observed genome equivalents within the panel and in naturally infected samples (Fig. 1), we also considered sensitivity within the samples spiked with the four lowest concentrations of M. bovis separately (DSeLC) (Table 2). The quantity of M. bovis genome equivalents from these four lowest-spiked samples were most similar to the quantities found in positive wild badger feces in previous work (37, 38). For Fast24-qPCR and Fast96-qPCR, sensitivity is dependent on DNA concentration, and estimating sensitivity from samples that contain M. bovis cell concentrations higher than those found in natural positive feces will produce a biased overestimate of true sensitivity. Despite the intention for spiked cell concentrations to cover a range similar to that found in naturally infected samples, it was clear that many of the spiked samples had substantially higher cell concentrations; hence the need for this subanalysis. We ascribe this discrepancy to the difference between cell number as measured by genome equivalents and that determined by CFU. Statistical comparisons of DSe with DSeLC were one tailed, as, *a priori*, we anticipated sensitivity to be lower at lower spiked M. bovis concentrations.

**TABLE 2:** DSe, DSeLC, and DSp

Test	Value (%) (95% CI, *n*)^*a*^		
	DSe	DSeLC	DSp
Fast24-qPCR (1st operator)	96.7ab* (94.5–99.0, 244)	89.2* (81.7–96.7, 65)	99.0 (97.8–100, 292)
Fast24-qPCR (2nd operator)	89.8a (86.0–93.6, 245)	87.7 (79.7–95.7, 65)	96.9 (95.0–98.9, 293)
Fast96-qPCR	88.4b* (84.3–92.4, 241)	75.0* (64.5–85.5, 64)	97.0 (95.0–98.9, 231)

a The letters “a” and “b” indicate pairwise comparisons within DSe: *P* < 0.05 by two-tailed Fisher’s exact test (Bonferroni corrected). Asterisks indicate pairwise comparisons between DSe and DSeLC: *P* < 0.05 by one-tailed Fisher’s exact test (Bonferroni corrected).

At the level of the social group, test performance was estimated by calculating herd sensitivity and specificity (HSe and HSp, respectively). These epidemiological terms refer to the ability of the tests to accurately identify positive social groups (“herds”) in the case of herd sensitivity and to accurately identify negative social groups in the case of herd specificity. As it is difficult to link feces to the animal which excreted them, positive test results can infer positivity only at the social-group level. The performance of a test at a social-group level is dependent on its sensitivity and specificity at an individual level, the number of samples tested (*n*), the true within-social-group prevalence (TP), and the threshold value of individual positives used to classify the social group as positive. Herd specificity is dependent on diagnostic specificity and the number of samples tested, and herd sensitivity (based on the binomial distribution) can be calculated from apparent within-social-group prevalence (AP), which is calculated as follows (45):

(1) AP = DSe × TP +[(1 − DSp) × (1 − TP)]

(2) $$\text{HSe}=1-\left(1-{\text{AP}}^{n}\right)$$

(3) $$\text{HSp}=1-{\text{DSp}}^{n}$$

The variance of apparent prevalence [Var(AP)] can be estimated as follows (46):

(4) $$\text{Var}\left(\text{AP}\right)\approx {\text{TP}}^{2}\times \frac{\text{DSe}\times \left(1-\text{DSe}\right)}{N}+{\left(1-\text{TP}\right)}^{2}\times \frac{\text{DSp}\times \left(1-\text{DSp}\right)}{M}$$

where DSe is estimated from *N* known positives and DSp is estimated from *M* known negatives. From this, 95% confidence intervals (CI) for apparent within-group prevalence (AP) can be calculated as follows:

(5) $$95\%\text{CI}\approx \text{AP}\pm 1.96\sqrt{\text{Var}\left(\text{AP}\right)}$$

The posttest probability at the social-group level, or the subjective probability of the presence of infection within a social group, can calculated as herd positive predictive value (HPPV) and herd negative predictive value (HNPV) from these estimates of social-group-level sensitivity and specificity, as follows (45):

(6) $$\text{HPPV}=\frac{\text{HSe × HP}}{\text{HSe × HP + }\left[\left(1-\text{HSp}\right)\times \left(1-\text{HP}\right)\right]}$$

(7) $$\text{HNPV}=\frac{\text{HSp}\times \left(1-\text{HP}\right)}{[\text{HSp}\times \left(1-\text{HP})\right]+\left[(1-\text{HSe}\right)\times \text{HP]}}$$

where herd prevalence (HP) is the proportion of social groups that contain individuals with disease. For the purposes of our analyses, we modeled *n* up to 20 as, based on our field experience, this represented a reasonable upper limit for unique samples taken over two sampling events (38). In addition, the range of HP (0.05 to 0.2) was chosen based on the range of prevalence within badger social groups (37, 38).

We modeled the effects of requiring two independent DNA extractions and qPCR tests to both give positive results (serial testing [47]) in order to assign a sample as positive. The equations for repeat diagnostic sensitivity and specificity (DSe^R^ and DSp^R^, respectively) are as follows:

(8) $${\text{DSe}}^{\text{R}}={\text{DSe}}^{\text{2}}$$

(9) $${\text{DSp}}^{\text{R}}=1-{\left(1-\text{DSp}\right)}^{2}$$

This is possible if samples are split and stored at the point of sampling or when introduced into the laboratory. False-positive results are likely to be the result of contamination with DNA extracted from other positive samples, most likely during the DNA extraction process, as our qPCR is 100% specific for M. bovis DNA (38). As such, reextraction from a second aliquot of feces would give an independent result.

We modeled the effects of using repeat extractions using Fast24-qPCR, as it was more sensitive and more specific (though not to a statistically significant degree).

Statistical analysis was performed in RStudio (48) using R (49). Graphics were created using ggplot2 (50).

## RESULTS

### Sensitivity and specificity at the sample level.

DNA was extracted from two replicates of a deidentified panel of badger feces containing known positive and negative samples using the Fast24 (panel 1) and Fast96 (panel 2) extraction methods, prior to qPCR screening and quantification. These panels’ identification was restored by APHA when all data had been collected. The performance of the two operators using the Fast24-qPCR method differed significantly in terms of DSe (Bonferroni-corrected *P* < 0.01; two-tailed Fisher’s exact test) (Table 2). DSeLC and DSp were not significantly different between the two operators, though both were lower for operator 2 (Op2). Operator 1 (Op1) possessed the most experience with the technique at the time of the study, which may explain the discrepancy.

DSe was significantly higher for the Fast24-qPCR method (Op1) than the Fast96-qPCR method (Bonferroni-corrected *P* < 0.01; two-tailed Fisher’s exact test, respectively) (Table 2), but this was not the case for Op2. DSeLC was not significantly different between Fast24-qPCR (Op1 versus Op2) or between Fast24-qPCR (either operator) and Fast96-qPCR. These comparisons of subsamples are comparatively statistically underpowered, however, though DSeLC was similar for both operators of Fast24-qPCR. Fast24-qPCR (Op1) and Fast96-qPCR DSe was significantly higher than DSeLC (*P* < 0.01 and *P* < 0.05, respectively; one-tailed Fisher’s exact test). This was not the case for Op2. Diagnostic specificity (DSp) was not significantly different between the two methods or between operators. For both methods, DSe meets the minimum threshold (20%) established prior to the study. In terms of DSp, Fast24-qPCR as performed by Op1 meets the minimum threshold (98%), though its 95% CI did drop below it; however, this is not the case for Op2, though the minimum threshold is within the 95% CI and the difference between operators is not statistically significant. Fast96-qPCR does not meet the threshold, though again the threshold is within the 95% CI.

### Sensitivity and specificity at the social-group level.

**FIG 2:**
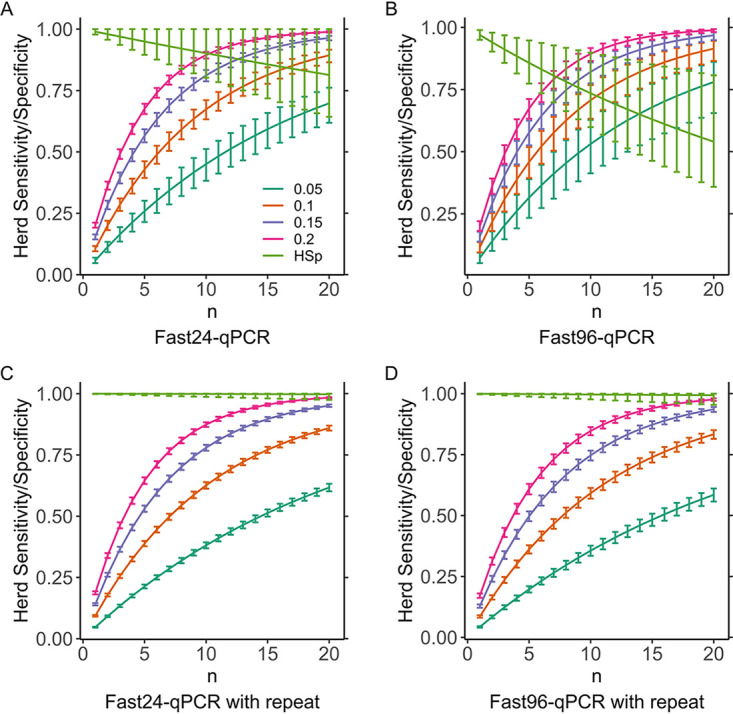
Relationship between herd sensitivity (HSe) and herd specificity (HSp) and the number of samples tested (*n*). A range of herd prevalences (HP) are modeled. HSe at a HP of 0.05 is shown in dark green, 0.1 in orange, 0.15 in purple, and 0.2 in pink, and HSp is shown in light green. (A) Fast24-qPCR; (B) Fast96-pPCR; (C) Fast24-qPCR with repeated positives; (D) Fast96-qPCR with repeated positives. For both Fast24-qPCR (A) and Fast96-qPCR (B), HSe increased with *n*; however, this came at the expense of HSp, which decreases with *n*. This can be alleviated by repeat testing of positives, which decreases the decline in HSp with *n* while maintaining HSe in both Fast24-qPCR (C) and Fast96-qPCR (D).

Group-level sensitivity (HSe) increased with number of samples and HP, while group-level specificity (HSp) decreased with number of samples for both Fast24-qPCR and Fast96-qPCR (Fig. 2A and B). To make this trade-off more favorable, two amendments were modeled, based on DSp and DSe from Op1. The first was serial testing, i.e., requiring independent, confirmatory re-extraction and qPCR retest of each positive feces to assign positive status to a sample. This substantially increased HSp while moderately decreasing HSe for both Fast24-qPCR and Fast96-qPCR (Fig. 2C and D). The second amendment, increasing the threshold of positive samples required to assign positive status to a social group from one to two, also increased HSp, but this had the effect of sharply decreasing HSe (Fig. S1).

The Fast24-qPCR data sets were also analyzed to model the effects of serial testing using both operators’ data sets as independent repeats (Table 3) in order to compare these to estimations based on DSe and DSp from Op1. When compared to the original DSe and DSp data (Table 2), sensitivity decreased (93.6% compared to 96.7%), while specificity increased (99.99% compared to 99.0%). DSe^R^ for the combined Fast24-qPCR data set was lower than estimated via equation 8 (87.3% compared to 93.6%; *P* < 0.05; two-tailed Fisher’s exact test), which was explained by the lower DSe for Op2. DSeLC^R^ and DSp^R^ for this combined data set are similar to the values estimated by equations 8 and 9 (78.5% compared to 79.6% and 100% compared to 99.99%, respectively). Repeat testing resulted in a substantially reduced decline in HSp caused by increasing sample number, thus allowing HSe to be increased without compromising HSp despite the reduction in DSe caused by repeat testing.

**TABLE 3:** DSe, DSeLC, and DSp with estimated effects of repeat testing and as measured by combining Fast24-qPCR panel results from both operators

Test	Value (95% CI, *n*)^*a*^		
	DSe (%)	DSeLC (%)	DSp (%)
Fast24-qPCR with repeat	93.6a (89.3–97.9)	79.6 (66.7–93.6)	99.99 (99.95–100)
Fast96-qPCR with repeat	85.5 (79.7–91.5)	66.9 (52.6–82.8)	99.97 (99.88–100)
Fast24-qPCR both operators	87.3a (83.1–91.5, 244)	78.5 (68.5–88.5, 65)	100 (100, 201)

a The letter “a” indicates pairwise comparison: *P* < 0.05 by two-tailed Fisher’s exact test.

### Predictive values at the social-group level.

**FIG 3:**
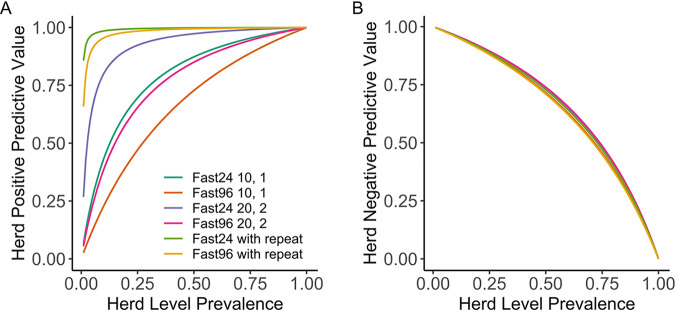
Relationship between herd positive predictive value (HPPV) (A), herd negative predictive value (HNPV) (B), and herd prevalence (HP)—the proportion of herds that are positive—for a variety of testing modalities. “Fast24-qPCR 10, 1” indicates Fast24-qPCR with 10 samples, with 1 positive sample required to designate the herd positive. Considering Fast24-qPCR 10, 1 and Fast96 10, 1 as the baseline, HPPV is improved by doubling both the number of samples tested, and the number of positive samples required, but not by as much as requiring the repeat testing of positive samples (10, 1 with repeat) (A). HNPV shows a similar relationship for all testing modalities (B).

At low levels of HP, both Fast24-qPCR and Fast96-qPCR have low HPPV but high HNPV (Fig. 2). HPPV is increased by testing 20 samples, with a threshold of 2 positives required to determine herd level infection, and is increased still further by requiring positive repeats in serial testing of positive samples (Fig. 3), which has a minimal effect on HNPV. These figures assume a within-herd shedding prevalence of 10%. The figures are based on estimates of HSe determined from DSeLC, though the values are similar if DSe is used. HPPV increases with sample number and HP, both with and without retesting of positives (Fig. S2). HNPV increases with sample number but decreases with HP, and the effect of retesting of positives on this is minimal (Fig. S3).

## DISCUSSION

The sensitivity and specificity of two tests that detect M. bovis in badger feces were estimated. The results presented here show that, on a per-sample basis, both Fast24-qPCR and Fast96-qPCR have diagnostic specificity that is similar or superior to that of existing trapping-based immunological tests. All tests met the threshold criteria for diagnostic sensitivity proposed in advance. Neither test consistently met the threshold criteria for diagnostic specificity, with only one operator of the Fast24-qPCR method meeting this threshold. However, we estimated that serial testing of positives would substantially increase specificity, and by combining the data sets from both operators, we showed 100% specificity. Such repeats show that Fast24-qPCR can be applied to multiple fecal samples from a social group in order to maximize group-level sensitivity without compromising group-level specificity. We predict that similar repeat tests could also improve the specificity of Fast96, though this remains to be demonstrated.

The Fast24-qPCR method, as performed by the first operator, was significantly more sensitive than the Fast96-qPCR, though this was not the case for the second operator, possibly due to the difference in experience between the two operators. Regardless, both extraction methods displayed high levels of sensitivity for the spiked samples analyzed in this study. However, for sensitivity, comparisons with other diagnostic tests should be applied with caution, as the two methods detect shedding, in contrast to immunological tests, which detect immune status. It is likely that there are more animals that are exposed to and show immunological responses to M. bovis than there are animals that actively shed the bacteria in their feces, and thus, a lower herd prevalence is expected for fecal testing than immunological testing, as previously shown by our laboratory (37). However, animals that are shedding may be both more infectious and more likely to spread infection via environmental contamination than seropositive animals that are not shedding. For this reason, and due to the difficulty of linking badger feces to individual animals, the tests cannot be used to determine the infection status of individuals, unless feces are taken directly from trapped animals.

For feces collected from latrines, the tests can be applied at a social-group level. To achieve adequate social-group-level sensitivity (HSe) requires the testing of multiple feces; however, this comes at the cost of decreasing group level specificity (HSp). This is overcome by the retesting of any positive samples using a previously stored aliquot of the same feces. This allows up to 20 fecal samples, approximately the upper limit for the quantity of unique samples that can be collected on two sampling trips, to be tested with low false-positive rates at the social-group level. Buzdugan et al. (33) have modeled HSe and HSp based on the parallel use of Stat-Pak and IFN-γ assays on samples from trapped badgers—i.e., both tests are used, and the animal is assigned positive status if either test is positive (47). Serial testing resulted in too-low DSe (30% at the individual-animal level) (33); therefore, to achieve the highest HSe and HSp, Buzdugan et al. (33) modeled the effects of parallel testing of animals, with a threshold requiring that two animals test positive for a social group to be considered bTB positive. Assuming that 50% of animals within a social group of 15 are trapped and tested, this results in an HSp of 91%, with an HSe of ∼60% at 25% prevalence of infection (33), though an HSp of >95% is also reported if 40% of the social group is trapped and tested. Serial testing of positive samples with Fast24-qPCR therefore shows a higher HSp than a trapping-based strategy. Given that initial data suggest that fecal qPCR can identify different animals than the IFN-γ and BrockTB StatPak assays (37), Fast24-qPCR could therefore be used to complement the immunological testing model described by Buzdugan et al. (33).

Fast24-qPCR is a noninvasive sampling method that can detect the shedding of M. bovis in badger feces at the level of the social group. When performed with retesting of positives, it has very high specificity and high sensitivity at the social-group level. In comparison to Fast24-qPCR, Fast96-qPCR increases the throughput of samples, but at the expense of reduced sensitivity and specificity. The reduction in specificity can likely be alleviated substantially with an independent retest of positive fecal samples using the Fast24-qPCR DNA extraction method, allowing testing of higher numbers of feces per social group, leading to higher sensitivity at the social-group level while maintaining high herd positive predictive value. While retesting of positives does increase the expense of the test, it need be applied only to the proportion of samples that are positive, which, based on previous research, we estimate to be in the range of 5 to 15% within regions where M. bovis is endemic. Given sufficient sampling effort, Fast24-qPCR therefore provides a social-group-level test that is capable of measuring the impacts of interventions designed to reduce the spread of bTB from badgers to cattle and vice versa, by accurately measuring the shedding of M. bovis into the environment.

## Supplementary Material

Supplemental file 1
