# Supplementary material for: Evaluation of a Fecal Shedding Test To Detect Badger Social Groups Infected with Mycobacterium bovis
Source: J Clin Microbiol. 2020 Dec 17;59(1):e01226-20. doi: 10.1128/JCM.01226-20 (PMC7771468; doi:10.1128/JCM.01226-20)
Supplement: Supplemental file 1 [file JCM.01226-20-s0001.pdf]

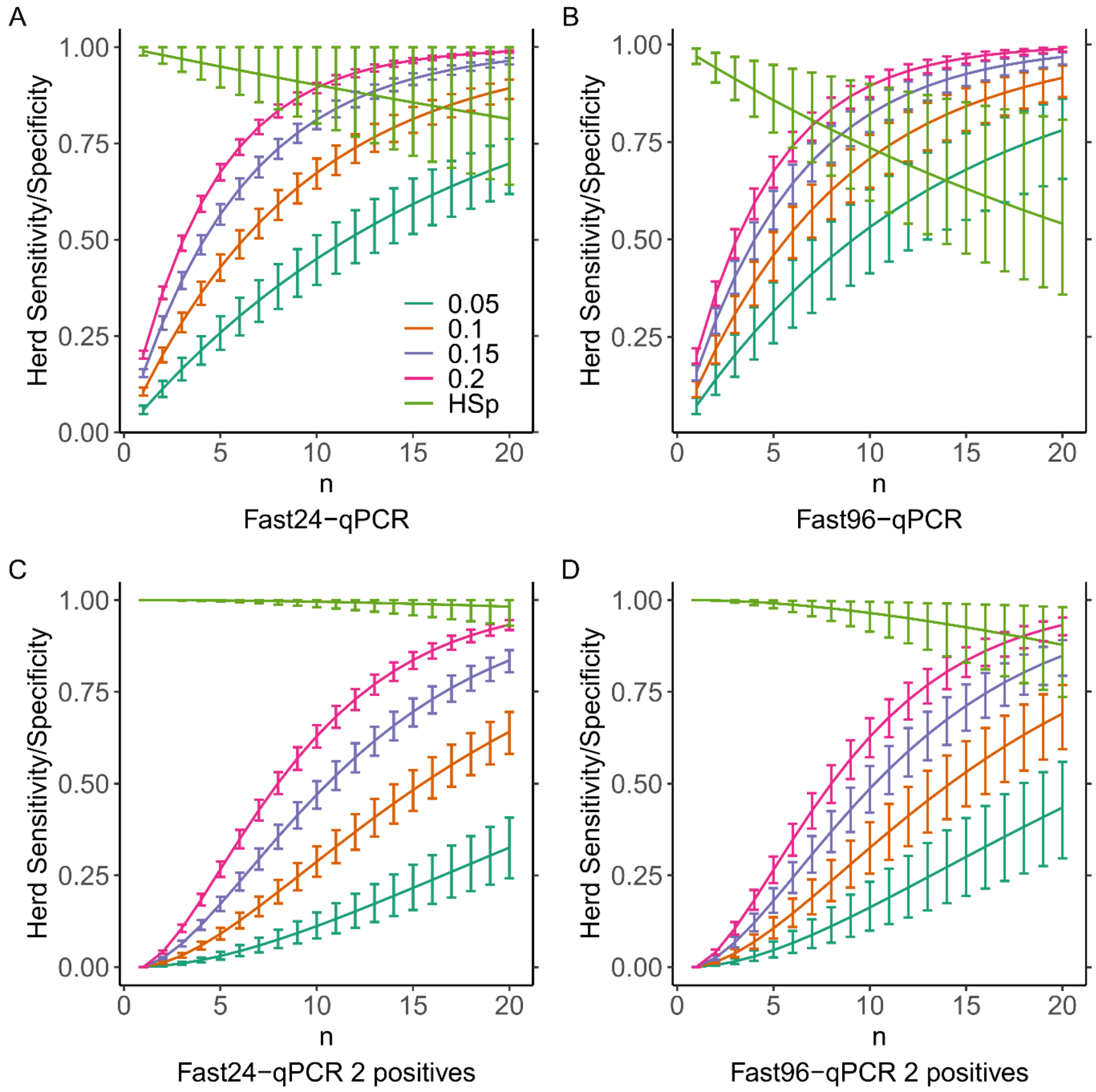

Figure S1

Relationship between Herd Sensitivity (HSe) and Herd Specificity (HSp), and the number of samples tested (n). A range of Herd Prevalences (HP) are modelled, from 0.05-0.2. HSe at HP 0.05 is shown in dark green, 0.1 (orange), 0.15 (purple), 0.2 (pink), and HSp is shown in light green. Fast24-qPCR (**A**), Fast96-qPCR (**B**), (**C**) and (**D**) show Fast24-qPCR and Fast96-qPCR respectively if 2 positive samples are required to determine herd posititivity. The higher sample positive threshold increases HSp but at the cost of decreasing HSe.

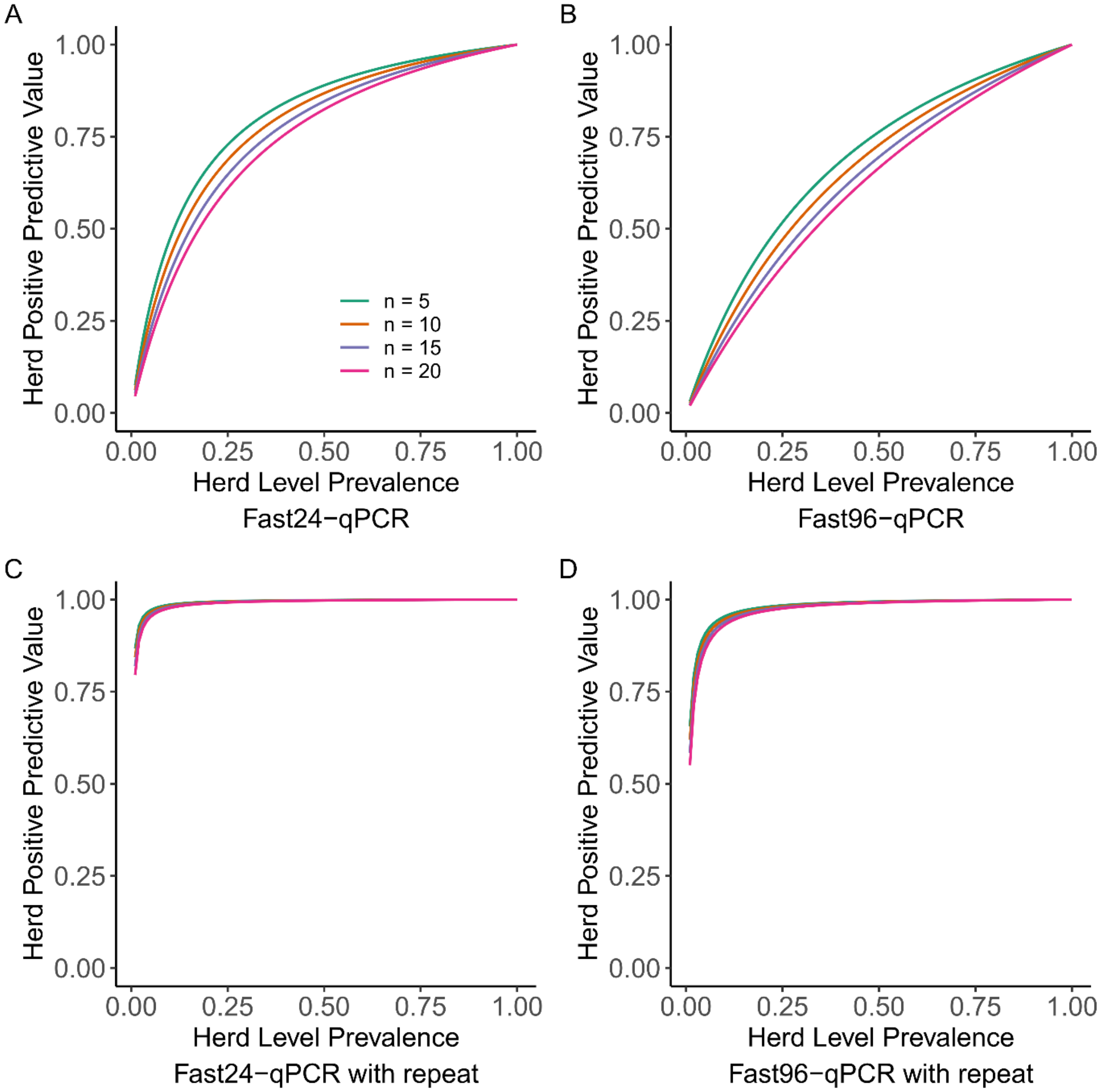

Figure S2.

Relationship between Herd Positive Predictive Value (HPPV) and number of samples used (n) over a gradient of Herd Level Prevalence (HP). n of 5 is shown in dark green, 10 (orange), 15 (purple), 20 (pink). Fast24-qPCR (**A**), Fast96-qPCR (**B**), Fast24-qPCR with repeats (**C**), Fast96-qPCR with repeats (**D**). For (**C**) and (**D**) repeats are performed with Fast24-qPCR. For both Fast24-qPCR and Fast96-qPCR HPPV decreases with increasing n, and requiring repeats reduces the effect of this relationship.

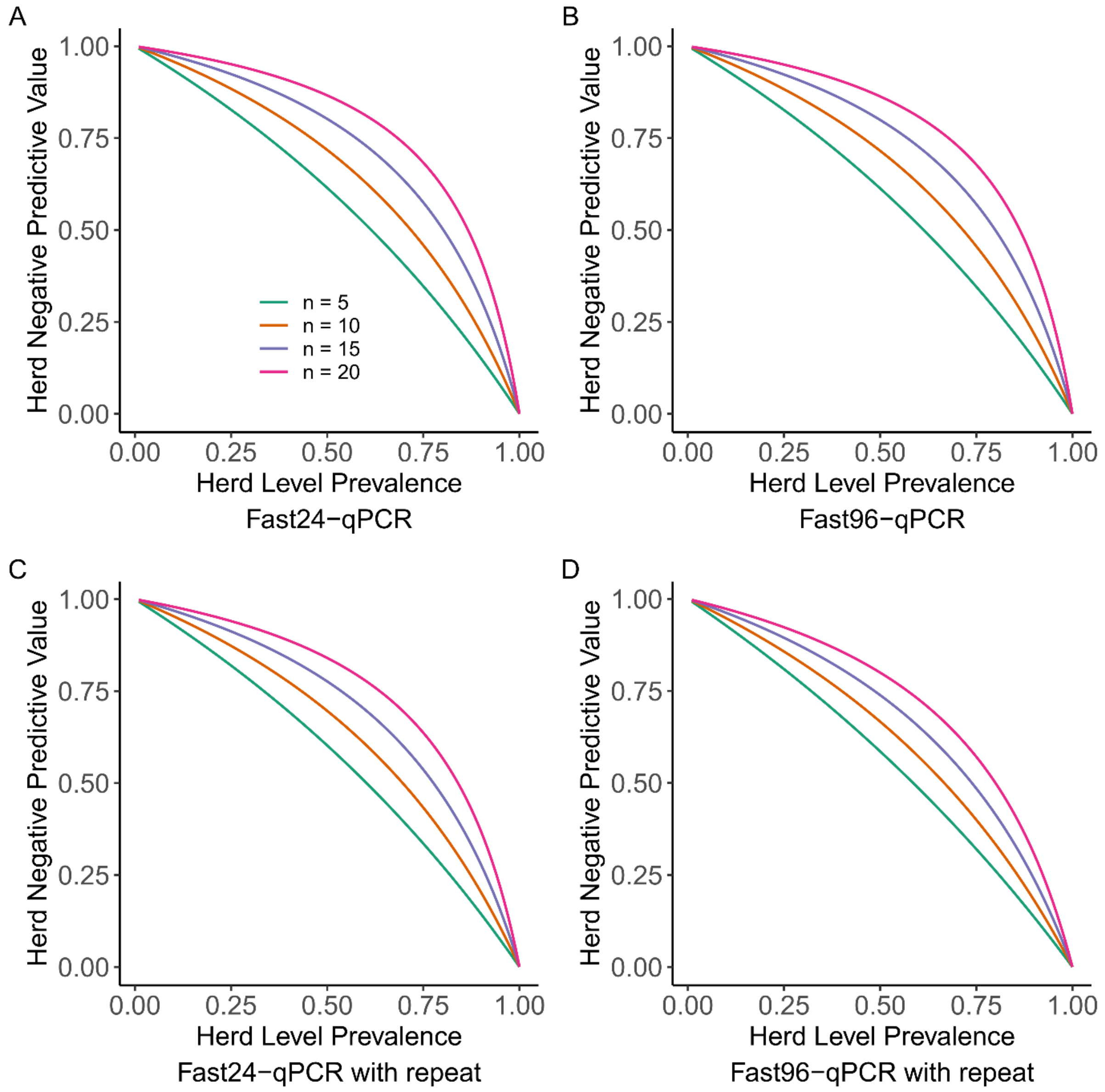

Figure S3.

Relationship between Herd Negative Predictive Value (HNPV) and number of samples used (n), over a gradient of Herd Level Prevalence (HP). n of 5 is shown in dark green, 10 (orange), 15 (purple), 20 (pink). Fast24-qPCR (**A**), Fast96-qPCR (**B**), Fast24-qPCR with repeats (**C**), Fast96-qPCR with repeats (**D**). For (**C**) and (**D**) repeats are performed with Fast24-qPCR. For both Fast24-qPCR and Fast96-qPCR HNPV increases with increasing n. Unlike HPPV, requiring repeats does not significantly alter this relationship.
